# Mitoriboscins: Mitochondrial-based therapeutics targeting cancer stem cells (CSCs), bacteria and pathogenic yeast

**DOI:** 10.18632/oncotarget.19084

**Published:** 2017-07-07

**Authors:** Bela Ozsvari, Marco Fiorillo, Gloria Bonuccelli, Anna Rita Cappello, Luca Frattaruolo, Federica Sotgia, Rachel Trowbridge, Richard Foster, Michael P. Lisanti

**Affiliations:** ^1^ Translational Medicine, School of Environment & Life Sciences, University of Salford, Greater Manchester, UK; ^2^ The Paterson Institute, University of Manchester, Withington, UK; ^3^ The Department of Pharmacy, Health and Nutritional Sciences, The University of Calabria, Cosenza, Italy; ^4^ Astbury Centre for Structural Molecular Biology, University of Leeds, West Yorkshire, UK; ^5^ School of Chemistry, Faculty of Mathematics and Physical Sciences, University of Leeds, West Yorkshire, UK

**Keywords:** antibiotic, drug design, mitochondrial ribosome, mitoribosome, mitochondria

## Abstract

The “endo-symbiotic theory of mitochondrial evolution” states that mitochondrial organelles evolved from engulfed aerobic bacteria, after millions of years of symbiosis and adaptation. Here, we have exploited this premise to design new antibiotics and novel anti-cancer therapies, using a convergent approach. First, virtual high-throughput screening (vHTS) and computational chemistry were used to identify novel compounds binding to the 3D structure of the mammalian mitochondrial ribosome. The resulting library of ∼880 compounds was then subjected to phenotypic drug screening on human cancer cells, to identify which compounds functionally induce ATP-depletion, which is characteristic of mitochondrial inhibition. Notably, the top ten “hit” compounds define four new classes of mitochondrial inhibitors. Next, we further validated that these novel mitochondrial inhibitors metabolically target mitochondrial respiration in cancer cells and effectively inhibit the propagation of cancer stem-like cells *in vitro*. Finally, we show that these mitochondrial inhibitors possess broad-spectrum antibiotic activity, preventing the growth of both gram-positive and gram-negative bacteria, as well as *C. albicans* - a pathogenic yeast. Remarkably, these novel antibiotics also were effective against methicillin-resistant Staphylococcus aureus (MRSA). Thus, this simple, yet systematic, approach to the discovery of mitochondrial ribosome inhibitors could provide a plethora of anti-microbials and anti-cancer therapies, to target drug-resistance that is characteristic of both i) tumor recurrence and ii) infectious disease. In summary, we have successfully used vHTS combined with phenotypic drug screening of human cancer cells to identify several new classes of broad-spectrum antibiotics that target both bacteria and pathogenic yeast. We propose the new term “mitoriboscins” to describe these novel mitochondrial-related antibiotics. Thus far, we have identified four different classes of mitoriboscins, such as: *1) mitoribocyclines, 2) mitoribomycins, 3) mitoribosporins and 4) mitoribofloxins*. However, we broadly define mitoriboscins as any small molecule(s) or peptide(s) that bind to the mitoribosome (large or small subunits) and, as a consequence, inhibit mitochondrial function, i.e., mitoribosome inhibitors.

## INTRODUCTION

Evidence is accumulating that increased mitochondrial biogenesis may play a critical role in the successful propagation and maintenance of the cancer stem-like cell (CSC) phenotype [1–9].

Analysis of transcriptional profiling data from human breast cancer samples (*N* = 28 patients) revealed that > 95 mRNA transcripts associated with mitochondrial biogenesis and/or mitochondrial translation are significantly elevated in cancer cells, as compared with adjacent stromal tissue [10, 11]. Remarkably, > 35 of these 95 upregulated mRNA’s encode mitochondrial ribosomal proteins (MRPs) [11]. MRPs are the functional subunits of the mitochondrial ribosomes (mitoribosomes), which are responsible for the mitochondrial translation of 13 protein components of the OXPHOS complex encoded by mitochondrial DNA. In this context, MRPS gene products are used to form the small subunit of the mitoribosome, while MRPL gene products are used to generate the large subunit of the mitoribosome [12–15].

Most of these 36 mitoribosome-related mRNA transcripts were elevated between 2- to 5-fold in human breast cancer cells, including seventeen members of the MRPS gene family (S7, S11, S12, S13, S14, S15, S17, S18A, S18B, S22, S26, S27, S28, S30, S31, S33, S35) and nineteen members of the MRPL gene family (L3, L9, L15, L16, L17, L18, L20, L22, L24, L33, L39, L40, L42, L46, L48, L49, L52, L54, L57) [11].

Proteomic analysis of human breast cancer stem-like cells also revealed the significant over-expression of several mitoribosomal proteins, such as MRPL45 and MRPL17, and 6 other proteins associated with mitochondrial biogenesis (HSPA9, TIMM8A, GFM1, HSPD1 [a.k.a., HSP60], TSFM, TUFM) [1]. Importantly, functional inhibition of mitochondrial biogenesis, using the off-target effects of certain bacteriostatic antibiotics, effectively ablated the propagation of CSCs, in 12 cell lines representing 8 different tumor types (breast, DCIS, prostate, ovarian, pancreatic, lung, melanoma and glioblastoma) [3, 5]. Virtually identical results were also obtained with *bonafide* OXPHOS inhibitors (pyrvinium pamoate and atovaquone), providing additional complementary evidence that functional mitochondria are required for the propagation of CSCs [3, 16]. Taken together, these preliminary studies provide the necessary evidence that the development of novel mitoribosome inhibitors might be a beneficial approach for the more effective treatment of cancer patients.

Recently, the 3D structures of both the large (39S) and the small (28S) subunits of the mammalian mitoribosome (55S) have been resolved [17–22], allowing for the rationale molecular design of mitoribosome inhibitors.

Here, we used the known 3D structure of the large 39S mammalian mitoribosome as a target to perform virtual high-throughput screening (vHTS). We coupled this computational chemistry approach with phenotypic drug screening, allowing for the functional identification and validation of novel compounds targeting mammalian mitoribosomes. The ability of these mitochondrial inhibitors to functionally prevent oxygen-consumption and halt ATP production was also demonstrated by metabolic flux analysis. Most importantly, these mitochondrial inhibitors effectively blocked the propagation of CSC, as predicted, providing proof-of-concept.

Interestingly, we also show that these mitochondrial inhibitors behave as broad-spectrum antibiotics, which is consistent with the well-established hypothesis that mitochondria originally evolved from the engulfment of aerobic bacteria, approximately 1.5 billion years ago [23–28]. This has important implications for more effectively combating the development of antibiotic-resistance.

## RESULTS

### Exploiting the evolutionary relationship between bacteria and mitochondria, to drive the discovery of new antibiotics and novel anti-cancer agents

**Figure 1:**
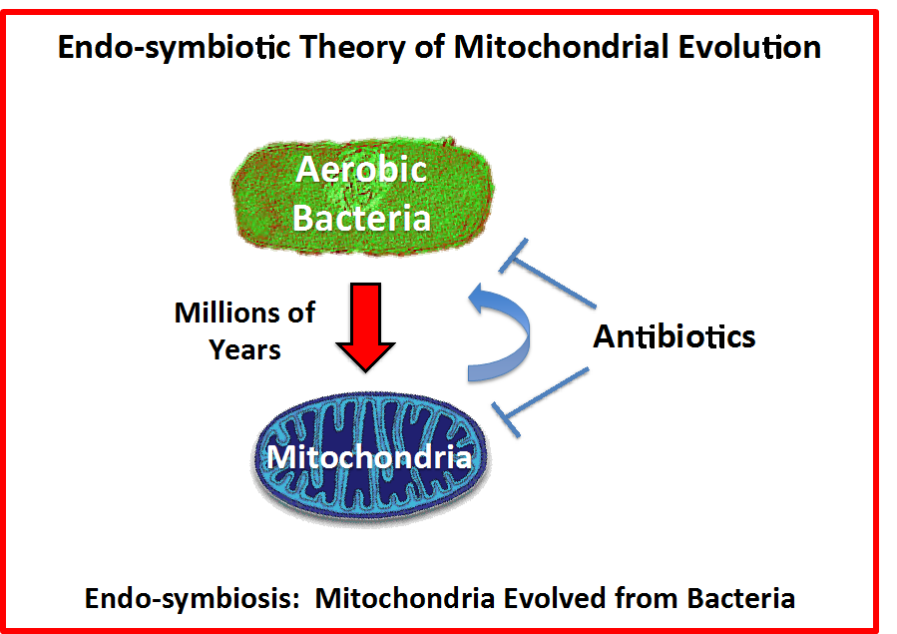
The endo-symbiotic theory of mitochondrial evolution: Implications for modern drug development Note that mitochondria originally evolved from engulfed aerobic bacteria, during millions of years of adaptation. A corollary of these findings is that many antibiotics also show mitochondrial side effects and effectively behave as inhibitors of mitochondrial protein translation (e.g., chloramphenicol, the tetracyclines and the erythromycins). Conversely, if we identify inhibitors of mitochondrial protein translation using mammalian cells, these drugs should also show anti-microbial activity, behaving as novel antibiotics. This would provide a new therapeutic strategy for efficiently generating novel drugs that target both mitochondria and bacteria, as well as pathogenic yeast strains.

The “Endo-symbiotic Theory of Mitochondrial Evolution” states that mitochondria originally evolved from aerobic bacteria that were incorporated into eukaryotic cells [23–28], during millions of years of adaptation (Figure 1). Consistent with this theory, we have recently shown that certain classes of well-known antibiotics that inhibit bacterial protein synthesis [29–31], can also be used to successfully target mitochondrial protein translation, especially in cancer stem-like cells (CSCs) [32].

However, the converse of these observations should also be true. More specifically, new inhibitors of mitochondrial protein translation should also possess anti-microbial activity. Here, to test this hypothesis directly, we used the known 3D structure of the mammalian mitochondrial ribosome (large subunit) to identify novel compounds that bind to it, in the context of virtual high-throughput screening (i.e., *in silico* drug screening). Once potential binding partners were identified *in silico*, then these 880 compounds were subjected to phenotypic drug screening *in vitro*, to select positive hits that functionally induced ATP-depletion in MCF7 human breast cancer cells. Approximately 85% of cellular ATP is normally generated by OXPHOS in mitochondria, so ATP-depletion is a valid surrogate marker for mitochondrial inhibition. However, only compounds depleting ATP levels without prominent cytotoxicity were selected for further analysis.

**Figure 2:**
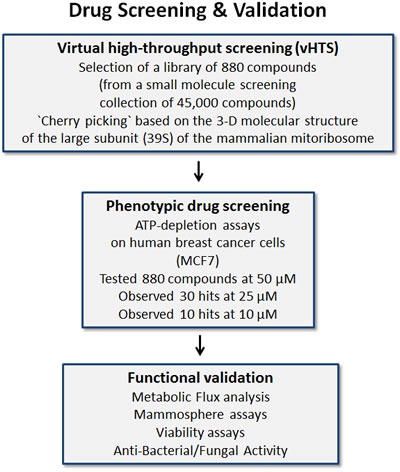
Schematic diagram illustrating our overall drug discovery strategy, employing both in silico drug discovery and phenotypic drug screening 1. Virtual high-throughput screening (vHTS) - We used the 3D structure of the large mammalian mitochondrial ribosome to screen a virtual collection of 45,000 compounds and identified a subset of 880 compounds that “bind” in silico. 2. Phenotypic drug screening - The resulting 880 compounds were then subjected to phenotypic drug screening at a concentration of 50 μM, to identify which compounds functionally induce ATP-depletion, before inducing cell death. Subsequently, positive hits were re-screened at lower concentrations (25 and 10 μM), to identify the top ten compounds that most potently induced ATP-depletion. 3. Functional validation - The top hits were then further validated using metabolic flux analysis, to determine specific effects on oxygen consumption, to estimate their anti-mitochondrial activity. Mammosphere assays (for assessing potential anti-cancer stem cell activity) and cell viability assays were also carried out. Finally, the top three compounds were assessed for anti-microbial activity, to determine their minimum inhibitory concentration (MIC) and they were compared side-by-side with known antibiotics.

These positive hits were then subjected to further validation, using the Seahorse metabolic flux analyzer, to confirm their mechanism of action as mitochondrial inhibitors. Finally, these novel compounds were tested on six distinct bacterial and/or yeast strains to investigate if they possess anti-microbial activity. This overall experimental strategy is outlined schematically as a flow-diagram in Figure 2.

### Identification and validation of novel inhibitors of the large mitochondrial ribosome

The resulting 880 compounds were first subjected to phenotypic drug screening at a concentration of 50 μM, to identify which compounds functionally induce ATP-depletion, before inducing cell death. Subsequently, positive hits were re-screened at lower concentrations (25 μM and 10 μM), to identify the top 10 compounds that most potently induced ATP-depletion.

**Figure 3:**
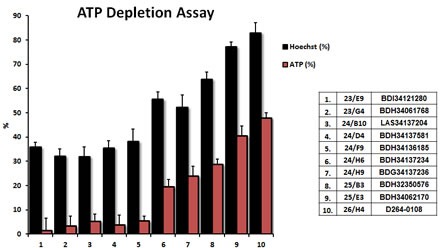
Comparison of the top 10 hits using the ATP-depletion assay The top ten hits were all evaluated at a concentration of 10 μM, to determine their rank order potency, for their ability to deplete ATP levels. MCF7 cells were treated with the selected compounds of at 10μM for 72 hours. Hoechst staining showed cell viability based on DNA staining, while measurement of ATP content revealed the effects of the compounds on metabolic activity. Compounds targeting the inhibition of metabolism were selected. Hoechst staining and ATP content were normalized to controls. Results are shown as mean ± SEM (n = 4).

Results from the ATP-depletion assay for the top 10 hits identified from phenotypic drug screening are shown in detail in Figure 3. Briefly, MCF7 cells were treated with the hit compounds at 10 μM for 72 hours. Hoechst staining showed cell viability based on DNA staining, while measurement of ATP content revealed the effect of compounds on metabolic activity during the very same treatments. Compounds inhibiting mitochondrial metabolism were selected for further analysis. Hoechst staining and ATP content were also normalized to controls. Results were rank-ordered, as indicated, based on their ability to effectively deplete ATP, without inducing overall cell toxicity - as reflected by maintenance of viability and cell attachment.

**Figure 4:**
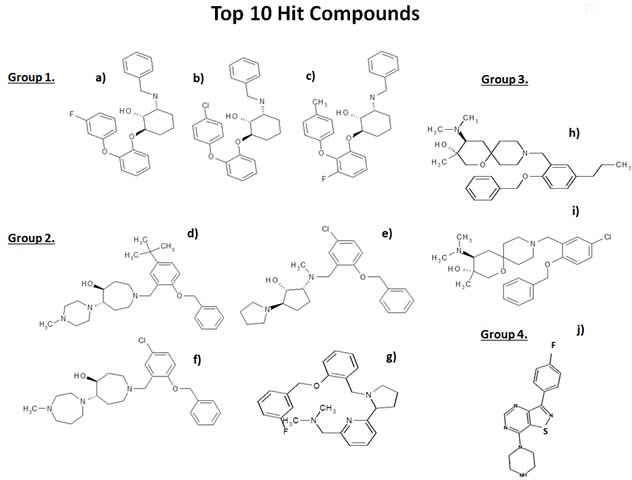
Chemical structures of the top 10 hits The most promising top 10 hits of the phenotypic drug screen were organized into 4 groups based on chemical structure: Group 1: a) 23/E9 (BDI34121280); b) 23/G4 (BDH34061768); and c) 25/E3 (BDH34062170). In this group, we focused on characterizing the activity of 23/G4. Group 2: d) 24/F9 (BDH34136185); e) 24/B10 (LAS34137204); f) 24/H6 (BDH34137234); and g) 25/B3 (BDH32350576). In this group, we focused on characterizing the activity of 24/F9. Group 3: h) 24/D4 (BDH34137581) and i) 24/H9 (BDG34137236). In this group, we focused on characterizing the activity of 24/D4. Group 4: j) 26/H4 (D264-0108).

Comparison of the chemical structures of these top 10 compounds identified 4 different drug classes, based on structural similarities, which are summarized in Figure 4. Three compounds fall into Group 1, four compounds in Group 2, two compounds in Group 3, and one compound in Group 4.

**Figure 5:**
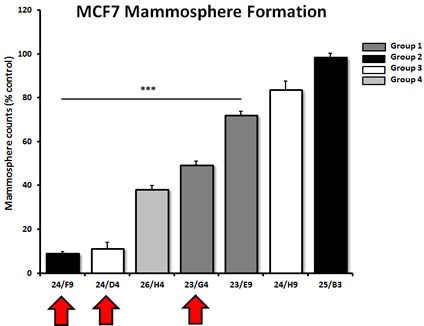
Effects of 7 top hit compounds on mammosphere formation, representing 4 different structural groups or classes Note that 5 of the 7 compounds evaluated significantly inhibited mammosphere formation, a measure of cancer stem cell activity, at a concentration of 5 μM. Compounds 24/F9, 24/D4 and 23/G4 were among the most effective, with an IC-50 < 5 μM. Results are shown as the mean ± SEM (n = 3). *** p < 0.001.

To validate the hypothesis that these compounds also target cancer stem-like cells (CSCs), the top 7 hits were compared in parallel for their ability to inhibit mammosphere formation in MCF7 cells. Importantly, 5 of the 7 compounds tested significantly inhibited mammosphere formation, at a concentration of 5 μM (Figure 5). For example, 23/G4 (Group 1) reduced mammosphere formation by 50% at this concentration. Similarly, 24/F9 (Group 2) and 24/D4 (Group 3), both reduced mammosphere formation by ∼90%.

**Figure 6:**
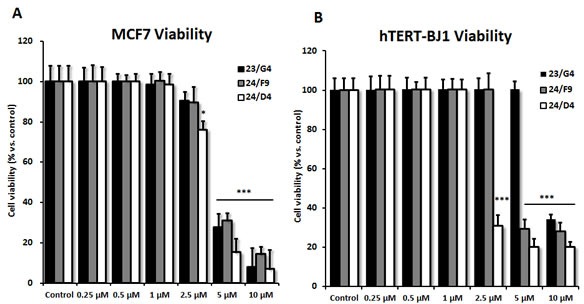
Effects of 3 top hit compounds on cell viability **A**. MCF7 human breast cancer cells. **B**. hTERT-BJ1 normal human fibroblasts. The SRB assay was performed after 72 hours of treatment. Note that 23/G4 has no effect on normal fibroblasts at 5 μM. However, at the same concentration, 23/G4 reduces the viability of MCF7 breast cancer cells by > 70%. Thus, 23/G4 shows a degree of selectivity for cancer cells. Results are shown as mean ± SEM (n = 6). *** p < 0.001, * p < 0.05.

Based on this analysis, we next selected 3 top hits to assess their functional effects on overall viability in MCF7 cell monolayers and normal human fibroblasts (hTERT-BJ1 cells) (Figure 6). Interestingly, 23/G4 (Group 1) reduced the viability of MCF7 cells by 70% at a concentration of 5 μM. However, 23/G4 had no effect on the viability of hTERT-BJ1 cells, when tested at the same concentration. Thus, it is possible to identify compounds, such as 23/G4, that preferentially target CSCs and “bulk” cancer cells, but not normal fibroblasts.

Next, we performed functional validation of the 3 top hits using the Seahorse Analyzer, to quantitatively measure oxygen consumption rate (OCR) and extracellular acidification rate (ECAR). OCR is a surrogate marker for OXPHOS and ECAR is a surrogate marker for glycolysis and L-lactate production.

**Figure 7:**
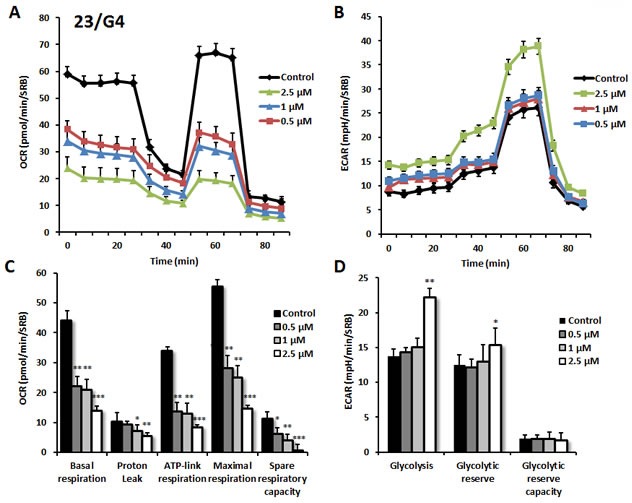
Effects of compound 23/G4 on the metabolic activity of MCF7 human breast cancer cells Oxygen consumption rate (OCR) and extracellular acidification rate (ECAR) was measured using the Seahorse XFe96 Metabolic Flux Analyzer. Then data were normalized to protein content (SRB assay). Note that 23/G4 treatment reduced mitochondrial respiration significantly even at a dose as low as 500 nM (see panel **A**.) by markedly decreasing basal and maximal respiration, as well as ATP production (panel **C**.). Treatment with the highest dose (2.5 μM) resulted in increased glycolysis (panels **B**., **D**.). MCF7 cells were treated with 23/G4 compound for 72 hours. Results are shown as mean ± SEM (n = 6). * p < 0.05, ** p < 0.01, *** p < 0.001.

**Figure 8:**
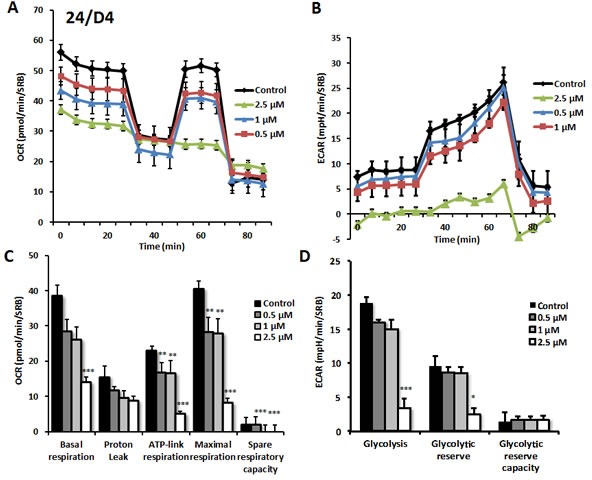
Effects of compound 24/D4 on metabolic activity of MCF7 human breast cancer cells Oxygen consumption rate (OCR) and extracellular acidification rate (ECAR) was measured using the Seahorse XFe96 Metabolic Flux Analyzer. Then data were normalized to protein content (SRB assay). Note that treatment with compound 24/D4 (2.5 μM) showed a marked inhibition of mitochondrial respiration (see panels **A**., **C**.). The same dose significantly reduced glycolysis (panels **B**., **D**.). MCF7 cells were treated with compound 24/D4 for 72 hours. Results are shown as mean ± SEM (n = 6). * p < 0.05, ** p < 0.01, *** p < 0.001.

**Figure 9:**
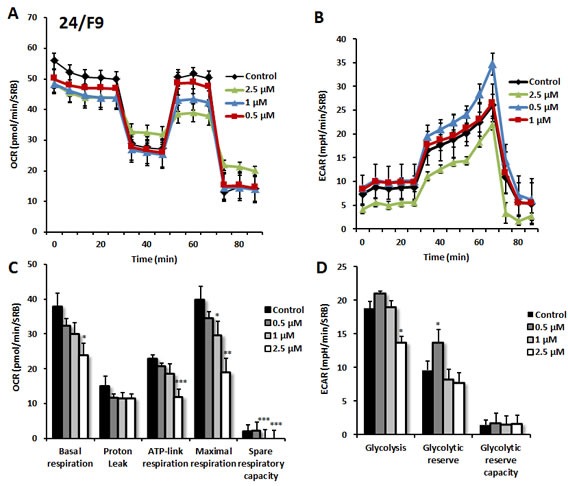
Effects of compound 24/F9 on metabolic activity of MCF7 human breast cancer cells Oxygen consumption rate (OCR) and extracellular acidification rate (ECAR) was measured using the Seahorse XFe96 Metabolic Flux Analyzer. Then data were normalized to protein content (SRB assay). Treatment with compound 24/F9 showed dose-dependent reduction of mitochondrial respiration, which was highest at 2.5 μM (panels **A**., **C**.). Inhibition of glycolysis was detected after treatment at the highest concentration (2.5 μM) of 24/F9. MCF7 cells were treated with compound 24/F9 for 72 hours. Results are shown as mean ± SEM (n = 6). * p < 0.05, ** p < 0.01, *** p < 0.001.

As predicted, 23/G4 (Group 1), 24/F9 (Group 2) and 24/D4 (Group 3) all dose-dependently inhibited mitochondrial oxygen-consumption in MCF7 cells, with 23/G4 being the most potent (Figures 7, 8 and 9). For example, 23/G4 reduced ATP levels by > 50% at a concentration of only 500 nM. In addition, 23/G4 reduced ATP levels by ∼75% at 2.5 μM (Figure 7). Remarkably, treatment with 23/G4, at the same concentrations, had little or no effect on the overall cell viability of MCF7 monolayers (Figure 6). Therefore, 23/G4 very effectively depleted ATP levels, without showing significant cytotoxicity.

**Figure 10:**
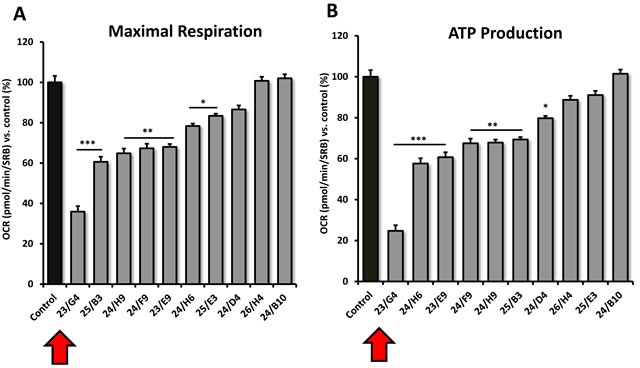
Comparative metabolic flux analysis of the top 10 hit compounds in MCF7 cells **A**. Maximal respiration. **B**. ATP production. Measurements were made after 48 hours of treatment of MCF7 cells with a given compound, at a concentration of 5 μM. Note that compound 23/G4 most significantly reduces both i) ATP production (by > 70%) and ii) maximal respiration (by > 60%). Oxygen consumption rates (OCR) were measured using the Seahorse XFe96 and then data were normalized for protein content (SRB assay). Maximal respiration and ATP-linked respiration data were calculated, normalized to control values and were plotted as percent control. Results are shown as mean ± SEM (n = 3). * p < 0.05, ** p < 0.01, *** p < 0.001.

Interestingly, 23/G4 induced an increase in glycolysis rates by > 1.5-fold, while 24/F9 and 24/D4 both suppressed glycolysis. This could explain why 24/F9 and 24/D4 were more potent than 23/G4 in the mammosphere assay, where 24/F9 and 24/D4 both reduced mammosphere formation by ∼90% at a concentration of 5 μM (Figure 5). The rank order potency of the top 10 hits for their ability to reduce i) maximal respiration and ii) ATP production is shown in Figure 10. Note that the top 6 compounds in this regard were 23/G4, 25/B3, 24/H9, 24/F9, 23/E9 and 24/H6, with 23/G4 being the most potent, yielding a > 75% reduction in ATP levels at 5 μM.

**Figure 11:**
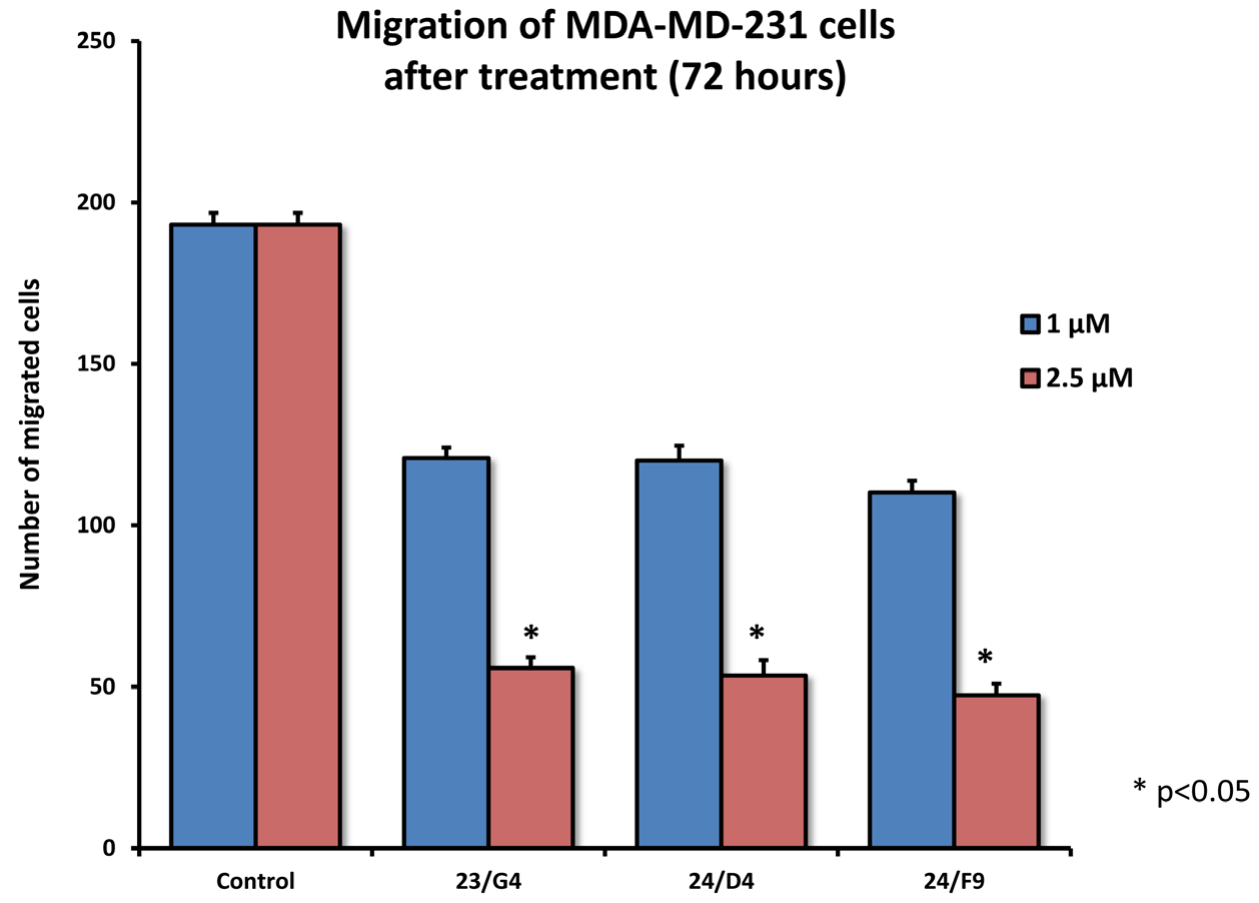
Effects of 3 top hit compounds on cell migration MDA-MB-231 cells were subjected to cell migration assays. 23/G4, 24/F9 and 24/D4 all significantly reduced cell migration, at concentrations of 1 and 2.5 μM. More specifically, 23/G4, 24/D4 and 24/F9 inhibited cell migration by > 70%, at a concentration of 2.5 μM.

As EMT and cell invasion are phenotypic features associated with “stemness” and distant metastasis [33–35], we also evaluated the effects of these compounds on the ability of another more aggressive breast cancer cell line, namely MDA-MB-231, to undergo cell migration. Figure 11 shows that 23/G4, 24/D4 and 24/F9 all inhibited cell migration by > 70%, at a concentration of 2.5 μM.

In summary, 23/G4 (Group 1) appears to be the most promising new lead compound, as it is more selective at targeting CSCs and cancer cells, while sparing normal cells (Figure 6). Also, 23/G4 is the most potent hit compound that effectively reduces mitochondrial ATP levels and induces glycolysis. Most importantly, our results provide the necessary proof-of-concept that new mitochondrial inhibitors can be rapidly developed, by combining *in silico* drug design with phenotypic drug screening.

### Novel mitochondrial inhibitors function as broad-spectrum antibiotics

As mitochondria originally evolved from aerobic bacteria, over millions of years of evolution, we speculated that these new mitochondrial inhibitors would also behave as novel antibiotics.

To test this hypothesis directly, we selected 3 top hit compounds (24/F9, 24/D4 and 23/G4) and examined their anti-microbial activity against two gram-positive bacterial strains (*Staph. aureus and Strep. pyogenes*) and three gram-negative bacterial strains (*E. coli, P. aeruginosa, K. pneumoniae*), as well as the pathogenic yeast strain *C. albicans*.

The inhibition ratios of the selected compounds, against pathogenic strains, were calculated by serial dilution. The average of three inhibition zone diameter measurements was compared against commercial drugs, using the Kirby-Bauer method.

Final interpretation of the measurements enabled all of the bacterial strains tested to be grouped into three categories (Sensitive, Intermediate and Resistant) as summarized in Tables 1 and 2. No growth inhibition was seen in the control (DMSO) (data not shown). Tables 1 and 2 illustrate that all five bacterial strains tested are sensitive to the 3 hit compounds (24/F9, 24/D4 and 23/G4).

**Table 1:** Gram-Positive Bacterial Antibiotic Sensitivity

Susceptibility Testing Gram-positive		*Staph. aureus* ATCC 25923			*Strept. pyogenes* ATCC 19615		
ANTIBIOTIC/INHIBITOR	CONTENT μg	EVALUATION					
		S	I	R	S	I	R
**Ciprofloxacin**	**4**	X			X		
**Rifampicin**	**4**	X				X	
**23/G4**	**8**	X			X		
**Gentamicin**	**8**	X			X		
**Tobramycin**	**8**	X				X	
**Levofloxacin**	**8**	X			X		
**Pefloxacin**	**8**	X			X		
**Azithromycin**	**8**	X				X	
**Clarithromycin**	**8**	X				X	
**Erythromycin**	**8**	X				X	
**Miocamycin**	**8**			X			X
**Roxithromycin**	**8**	X				X	
**Co-trimoxazole**	**8**	X			X		
**Amoxicillin / Clavulanic acid**	**8/4**		X			X	
**Piperacillin**	**16**	X			X		
**24/D4**	**32**	X			X		
**Netilmicin**	**32**	X			X		
**Cefaclor**	**32**		X			X	
**Cefixime**	**32**	X				X	
**Cefonicid**	**32**	X			X		
**Ceftazidime**	**32**	X			X		
**Cefuroxime**	**32**	X			X		
**Ampicillin / Sulbactam**	**32/16**		X		X		
**24/F9**	**64**	X			X		
**Ceftriaxone**	**64**	X			X		
**Fosfomycin**	**200**	X				X	

The anti-bacterial activity of three hit compounds (24/F9, 24/D4 and 23/G4) was compared to known antibiotics, across two different gram-positive bacterial strains (Staph. aureus and Strep. pyogenes). S, sensitive; I, intermediate; R, resistant.

**Table 2:** Gram-Negative Bacterial Antibiotic Sensitivity

Susceptibility Testing Gram-negative		*E. coli* ATCC 25922			*P. auriginosa* ATCC 27853			*K. pneumoniae* ATCC 13883		
ANTIBIOTIC/INHIBITOR	CONTENT μg	EVALUATION								
		S	I	R	S	I	R	S	I	R
**Ciprofloxacin**	**4**	X			X			X		
**Rifampicin**	**4**		X			X			X	
**Gentamicin**	**8**	X			X			X		
**Tobramycin**	**8**		X		X			X		
**Lomefloxacin**	**8**	X				X			X	
**Levofloxacin**	**8**	X			X			X		
**Pefloxacin**	**8**	X			X			X		
**Co-trimoxazole**	**8**	X			X			X		
**24/D4**	**32**	X			X			X		
**23/G4**	**32**		X		X			X		
**Amikacin**	**32**	X			X			X		
**Ceftazidime**	**32**	X			X			X		
**Cefuroxime**	**32**	X			X			X		
**Nalidixic acid**	**32**		X		X			X		
**Teicoplanin**	**32**	X				X			X	
**Aztreonam**	**32**	X			X			X		
**Amoxicillin / Clavulanic acid**	**32/16**		X		X			X		
**Ampicillin / Sulbactam**	**32/16**	X				X			X	
**Cefotaxime**	**64**	X			X			X		
**24/F9**	**64**	X				X		X		
**Cefoperazone**	**64**	X			X			X		
**Cefotaxime**	**64**	X			X			X		
**Ceftriaxone**	**64**	X			X			X		
**Nitrofurantoin**	**128**	X			X			X		
**Piperacillin/Tazobactam**	**128/4**	X			X			X		
**Ticarcillin/Clavulanic acid**	**128/4**	X			X			X		
**Fosfomycin**	**200**		X		X			X		

The anti-bacterial activity of three hit compounds (24/F9, 24/D4 and 23/G4) was compared to known antibiotics, across three different gram-negative bacterial strains (E. coli, P. aeruginosa, K. pneumoniae). S, sensitive; I, intermediate; R, resistant.

Therefore, in order to determine the minimal inhibitory concentration (MIC) for 24/F9, 24/D4 and 23/G4, the broth dilution method was performed. As expected, the MIC determination results were in good agreement with the disc-diffusion susceptibility test. Table 3 shows the MIC determination results obtained as compared to known antibiotics, against the tested bacterial strains and *C. albicans*. Importantly, note that compound 23/G4 showed the greatest broad-spectrum activity and potency, as compared with compounds 24/F9 and 24/D4.

**Table 3:** Minimum Inhibitory Concentrations (MIC): 5 Bacterial Strains and 1 Pathogenic Yeast

Minimum inhibitory concentration (compare with common antibiotics)	MIC μg/ml											
ANTIBIOTIC/INHIBITOR	*E. coli* ATCC 25922		*P. auriginosa* ATCC 27853		*K. pneumoniae* ATCC 13883		*Staph. aureus* ATCC 25923		*Strept. pyogenes* ATCC 19615		*C. albicans* ATCC 13883	
	50%	99%	50%	99%	50%	99%	50%	99%	50%	99%	50%	99%
**24/D4**	16	32	16	32	16	32	16	32	16	32	-	>64
**23/G4**	>16	>32	16	32	16	32	4	8	4	8	8	16
**24/F9**	32	64	>32	>64	32	64	32	64	32	64	-	>64
**DOXYCYCLINE**	0.5	2	1	2	2	4	0.5	1	0.5	1	-	>64
**LINEZOLID**	128	>128	64	256	128	256	1	2	1	2	-	-
**AMOXICILLIN**	16	>32	128	256	32	>64	4	8	4	8	-	-
**MICONAZOLE**	-	-	-	-	-	-	-	-	-	-	0.5	1

The anti-microbial activity of three hit compounds (24/F9, 24/D4 and 23/G4) was compared to known antibiotics, using five different bacterial strains (Staph. aureus, Strep. pyogenes, *E. coli, P. aeruginosa, K. pneumoniae*) and one pathogenic yeast/fungus (*C. albicans*). Note that compound 23/G4 showed the greatest broad-spectrum activity and potency, as compared with compound 24/F9 and 24/D4.

Finally, Table 4 shows that Methicillin-resistant Staphylococcus aureus (MRSA) is also sensitive to 23/G4 and 24/D4. We confirmed that this strain of MRSA was indeed resistant to amoxicillin, as predicted. Thus, it is possible to successfully use this new drug discovery strategy employing human cancer cells, to isolate new antibiotics that can target drug-resistant bacteria, such as MRSA.

**Table 4:** Minimum Inhibitory Concentrations (MIC): MRSA versus MSSA

Minimum inhibitory concentration (compare with common antibiotics)	MIC μg/ml			
ANTIBIOTIC/INHIBITOR	MRSA ATCC 43300		MSSA ATCC 25923	
	50%	99%	50%	99%
**24/D4**	16	>32	16	32
**23/G4**	16	>32	4	8
**24/F9**	64	>64	32	64
**AMOXICILLIN**	>64	>64	4	8

The anti-microbial activity of three hit compounds (24/F9, 24/D4 and 23/G4) was compared to known antibiotics, using two different bacterial strains of *Staph. aureus*, MRSA (methicillin-resistant *Staph. aureus*) and MSSA (methicillin-sensitive *Staph. aureus*). Note that MRSA is resistant to amoxicillin, as expected.

## DISCUSSION

### Discovery of the mitoriboscins: targeting the mitochondrial ribosome

**Figure 12:**
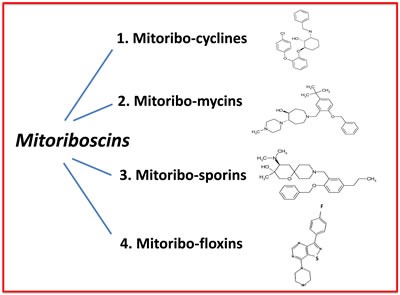
Mitoriboscins: Four new classes of mitochondrial inhibitors The structures of 23/G4, 24/F9, 24/D4 and 26/H4 are shown, which now define 4 newly discovered classes of small molecules. These novel molecules were identified because of their structural and functional mitochondrial inhibitory activity in human cancer cells. As such, this convergent screening approach will undoubtedly yield other classes of new antibiotics, as well novel anti-cancer agents. We specifically define mitoriboscins as any small molecule that binds to the mitoribosome (large or small subunits); this binding activity ultimately inhibits mitochondrial function.

Here, we have used state-of-the art computational chemistry to select novel compounds that bind to the 3D structure of the large subunit of the mammalian mitochondrial ribosome. Out of the 45,000 compounds tested, approximately 880 showed promising results, yielding a hit rate of 2%. To validate their functional ability to target mitochondria *in vivo*, we next performed phenotypic drug screening in human breast cancer cells (MCF7 cells). Using this approach, we selected the top 10 hit compounds (10/880 = 1%) that effectively depleted cellular ATP levels. These top 10 hit compounds were then subjected to further validation using the Seahorse XFe96, to measure mitochondrial oxygen consumption and glycolysis. This approach allowed us to rank-order these top 10 hits, identifying the compound 23/G4 as the most potent (1/45,000 = 0.00002 = 0.002 %). Notably, 23/G4 inhibited mitochondrial ATP production in MCF7 cells, with an IC-50 of approximately 500 nM. Remarkably, 23/G4 showed no cytotoxicity against normal human fibroblasts, even at a concentration (5 μM) that reduced cancer cell viability by > 70% (Figure 6). Importantly, the top hit compounds that we identified also potently inhibited CSC propagation and cancer cell migration, all in the low micro-molar range. Finally, we also showed that 3 of these top hit compounds also behave as antibiotics, inhibiting the growth of pathogenic bacteria and yeast. Importantly, 23/G4 and 24/D4 were also effective against MRSA. Thus, we propose the new term mitoriboscins, to describe these mitochondrial-related antibiotics. Moreover, the 4 classes of mitoriboscins that we describe here, we have designated as 1) mitoribocyclines, 2) mitoribomycins, 3) mitoribosporins and 4) mitoribofloxins (Figure 12). We generally define mitoriboscins as any small molecule that binds to the mitoribosome (large or small subunits) and, as a consequence, inhibits mitochondrial function.

**Figure 13:**
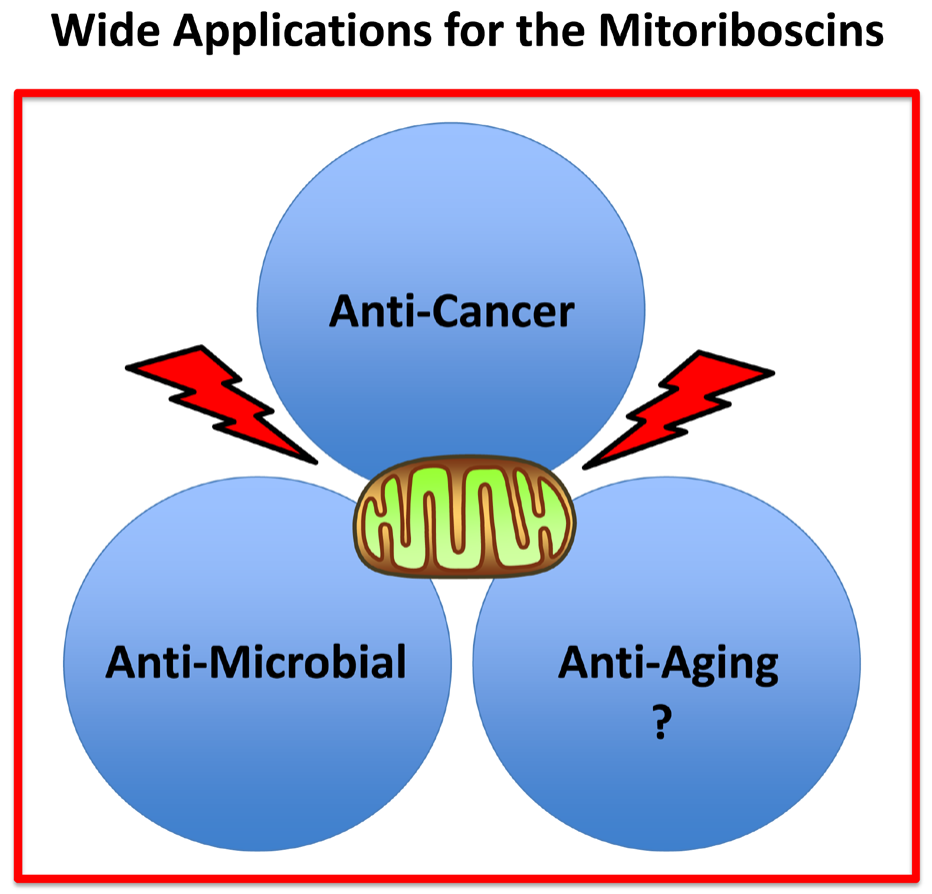
Practical uses of the mitoriboscins: Targeting mitochondria We propose that mitoriboscins will be therapeutically useful for the treatment of a variety of human diseases, including cancers and infectious illnesses, caused by pathogenic bacteria and fungi. In addition, we speculate that mitoriboscins may be helpful in the context of chronological aging, for either extending lifespan or healthspan. Thus, mitoriboscins will have anti-cancer, anti-microbial and anti-aging properties.

Although the novel compounds that we identified here are relatively potent, we envision that further modifications will be required to better optimize their ability to i) disrupt mitochondria in CSCs and ii) to halt the growth of micro-organism(s), for the treatment of oncologic and infectious diseases respectively. Nevertheless, our results provide the necessary proof-of-principle that it is possible to identify new antibiotics to target pathogenic bacteria and yeast, by employing ATP production in human cancer cells as a simple phenotypic screening tool (Figure 13).

### The mitoribosins: therapeutic use to combat ageing and extend healthspan

Interestingly, genetic inhibition of mitochondrial protein translation has also been shown to have beneficial “side-effects”, such as the effective slowing of the aging process and increased lifespan in model organisms. Houtkooper et al., 2013, showed that lower steady-state levels of Mrps5 (a mitoribosomal protein) were strongly functionally correlated with longer murine lifespan, resulting in a significant increase of ∼250 days [36]. In addition, selective knock-down of Mrps5 in *C. elegans* dramatically increased lifespan. Importantly, Mrps5 knock-down worms showed significant decreases in mitochondrial respiration and ATP production [36]. Similarly, knock-down of the worm homologs of mitochondrial complex I, III, IV and V, as well as several TCA cycle enzymes, all robustly extended lifespan, further implicating reduced OXPHOS activity and lower ATP levels as the mechanism [37–39]. Finally, pharmacological inhibition of mitochondrial biogenesis (using the off-target side-effects of doxycycline), also significantly increased lifespan in *C. elegans* [36]. Thus, lower doses of the mitoriboscins could potentially be used to therapeutically target the aging process, to extend healthspan (Figure 13). Further studies will be required to explore this intriguing area of investigation.

## MATERIALS AND METHODS

### Materials

MCF7 and MDA-MB-231 cells were purchased from the ATCC (American Type Culture Collection). Gibco-brand cell culture media (DMEM and DMEM/F12) was purchased from Life Technologies. The top 6 hit compounds were custom-synthesized in larger quantities by ASINEX Corp., and compound D264-0108 (26/H4) was custom-synthesized by ChemDiv Inc.

### Bacterial and fungus strains

The strains *Escherichia. coli* (ATCC 25922), *Klebisella pneumoniae* (ATCC 13883), *Pseudomonas aeruginosa* (ATCC 27853), *Staphylococcus aureus MSSA* (ATCC 25923), *Staphylococcus aureus MRSA* (ATCC 43300), *Streptococcus pyogenes* (ATCC 19615) and *Candida Albicans* (ATCC 13883) were provided by Remel Microbiology (Thermo Fisher). The cells were grown in Müller-Hinton broth II (MHB; Difco, Detroit, MI, USA) containing 2g/l beef infusion solids, 17.5 g/l casein hydrolysate, 1.5 g/l starch. The final pH was adjusted to 7.4.

### Virtual high-throughput screening (vHTS)

Compounds were selected from a small molecule screening collection of 45,000 compounds. Initial virtual high-throughput screening (vHTS) used the eHiTS screening program [40] to identify the top 5,000 ranked compounds based on predicted binding affinity to the large subunit (39S) of the mammalian mitoribosome [21]. To efficiently perform the docking, a series of clip files was prepared spanning the entire protein structure and the virtual library docked at each of the clip files. Consensus scoring of these top 5,000 compounds was carried out using AutoDock 4.2 [41], based on using the same general binding site for each compound predicted from the eHiTS screen. A total of 880 compounds performing well in these analysis steps were then selected for phenotypic drug screening.

### Phenotypic drug screening, with a metabolic ATP-depletion assay

MCF7 cells (6,000 cells/well) were plated into black clear-bottom 96-well plates and incubated overnight before treatment. The resulting 880 compounds first identified by vHTS were then subjected to phenotypic drug screening at a concentration of 50 μM, to identify which compounds functionally induce ATP-depletion, before inducing cell death. Subsequently, positive hits were re-screened at lower concentrations (25 μM and 10 μM), to identify the top 10 compounds that most potently induced ATP-depletion. Compounds were tested after 72 hours of incubation and experiments were performed in duplicate. After treatment, media was aspirated from the wells and plates were washed with warm PBS (supplemented w/Ca2+ and Mg2+). Then, cells were incubated with a Hoechst 33342 (Sigma) staining solution (10 μg/ml) for 30 min and washed with PBS (to estimate cell viability). Fluorescence was read with a plate reader using excitation/emission wavelenghts at 355/460-nm. Then, the CellTiter-Glo luminescent assay (Promega) was performed to measure metabolic activity (ATP content) in the very same wells that were treated with a given compound. Assays were performed according to the manufacturer’s protocol. Fluorescence intensity (Hoechst staining) and luminescence intensity (ATP content) was normalized to vehicle-alone treated controls and were displayed as percent control for comparison.

### Cell viability assay

The Sulphorhodamine (SRB) assay is based on the measurement of cellular protein content. After treatment for 72h in 96-well plates, cells were fixed with 10% trichloroacetic acid (TCA) for 1h in the cold room, and were dried overnight at room temperature. Then, cells were incubated with SRB for 15 min, washed twice with 1% acetic acid, and air dried for at least 1h. Finally, the protein-bound dye was dissolved in a 10 mM Tris, pH 8.8, solution and read using the plate reader at 540-nm.

### Mammosphere formation assays

A single cell suspension of MCF7 cells was prepared using enzymatic (1x Trypsin-EDTA, Sigma Aldrich) and manual disaggregation (25 gauge needle) [42]. Cells were then plated at a density of 500 cells/cm2 in mammosphere medium (DMEM-F12/B27/20-ng/ml EGF/PenStrep) in non-adherent conditions, in culture dishes coated with (2-hydroxyethylmethacrylate) (poly-HEMA, Sigma). Cells were grown for 5 days and maintained in a humidified incubator at 37°C at an atmospheric pressure in 5% (v/v) carbon dioxide/air. After 5 days in culture, spheres > 50 μm were counted using an eye-piece graticule, and the percentage of cells plated which formed spheres was calculated and is referred to as percent mammosphere formation, normalized to vehicle-alone treated controls. Mammosphere assays were performed in triplicate and repeated three times independently.

### Seahorse XFe96 metabolic Flux analysis

Extracellular acidification rates (ECAR) and real-time oxygen consumption rates (OCR) for MCF7 cells were determined using the Seahorse Extracellular Flux (XF96) analyzer (Seahorse Bioscience, MA, USA). MCF7 cells were maintained in DMEM supplemented with 10% FBS (fetal bovine serum), 2 mM GlutaMAX, and 1% Pen- Strep. 5,000 cells per well were seeded into XF96-well cell culture plates, and incubated overnight at 37°C in a 5% CO2 humidified atmosphere. After 24h, cells were treated with the top three hit compounds at various concentrations (or vehicle alone). After 72h of treatment, cells were washed in pre-warmed XF assay media (for OCR measurement, XF assay media was supplemented with 10mM glucose, 1mM Pyruvate, 2mM L-glutamine and adjusted at pH 7.4). Cells were then maintained in 175 μL/well of XF assay media at 37°C, in a non-CO2 incubator for 1h. During incubation, 25 μL of of 80mM glucose, 9μM oligomycin, 1M 2-deoxyglucose (for ECAR measurement) and 25 μL of 10μM oligomycin, 9μM FCCP, 10μM rotenone, 10μM antimycin A (for OCR measurement) in XF assay media was loaded into the injection ports of the XFe-96 sensor cartridge. During the experiment, the instrument injected these inhibitors into the wells at a given time point, while ECAR/OCR was measured continuously. ECAR and OCR measurements were normalized by protein content (Sulphorhodamine B assay). Data sets were analyzed by XFe-96 software, using one-way ANOVA and Student’s *t*-test calculations. All experiments were performed in triplicate.

### Cell migration assay

The migration of MDA-MD-231 cells was carried out, essentially as we previously described, with minor modifications [43].

### Gram (+ve) and gram (−ve) susceptibility testing

The antimicrobial susceptibilities of the novel compounds were evaluated using the Kirby-Bauer disc-diffusion method [44–46], performed according to CLSI guidelines and results were interpreted using CLSI breakpoints [47–48]. Antibiotics disks against gram(+ve) and gram(−ve) bacterias (from Oxoid™) were used as positive controls. All new componds (24/D4, 24/F9, 23/G4) were prepared by dissolving them in dimethyl sulfoxide (DMSO, from Sigma/Aldrich Company; St. Louis, MO, USA) and were utilized to impregnate the Blank Antimicrobial Susceptibility Disks (Oxoid™). Specifically, overnight cultures of bacteria tested were adjusted to a turbidity of 0.5 McFarland standards (106 CFU/ml) before inoculation onto agar plates with sterile cotton swabs. A cotton swab dipped in the cell culture was streaked onto an agar plate surface in such a way as to obtain a uniform layer of bacteria across the whole surface. After 10-15 min, the antibiotics disks or novel compounds disks were laid on the inoculated surface of the agar plates; then, all agar plates were incubated at 37°C, overnight. The diameters of inhibition were measured and susceptibility was expressed in terms of resistance (R), moderate susceptibility (I) and susceptibility (S). Agar plates inoculated with bacteria tested with impregnated DMSO disks were used as controls. The result obtained on single bacteria strain was confirmed by Sensi test gram-positive and Sensi test-gram-negative kits (*Liofilchem s.r.l*.). Disc-diffusion susceptibility test was performed in triplicate and repeated three times independently.

### Anti-microbial activity and MIC values determination

The minimal inhibitory concentration (MIC) of the antibacterial compounds was determined using the broth dilution method, according to CLSI guidelines [48]. Briefly, a solution content the new compounds (or several antibiotics used as positive control) was diluted, serially, with MHB medium. Then, the suspensions of the microorganisms, prepared from overnight cultures of bacteria in the MHB medium, at a concentration of 106 cfu x ml-1, were added to each dilution in a 1:1 ratio McFarland standards were used as a reference to adjust the turbidity of microorganism suspensions. Growth (or lack thereof) of the microorganisms was determined after incubation for 24 h at 37°C by turbidimetry (wavelength of 600 nm). MIC 50 and MIC 99 were defined as the minimum inhibitory concentration of the compound required for 50% and 99% inhibition of bacterial growth [49, 50]. The negative control tubes did not contain bacterial inoculum while the positive control tubes containing only DMSO, were antibiotics or compounds free. The susceptibility test by measurement of MIC was performed in triplicate and repeated three times independently.

### Statistical analyses

Statistical significance was determined using the Student’s *t*-test, values of less than 0.05 were considered significant. Data are shown as the mean ± SEM, unless stated otherwise.
